# Cardiac fibroblasts in heart failure and regeneration

**DOI:** 10.3389/fcell.2024.1388378

**Published:** 2024-04-18

**Authors:** Alenca Harrington, Thomas Moore-Morris

**Affiliations:** Institut de Génomique Fonctionnelle, University of Montpellier, CNRS, INSERM, Montpellier, France

**Keywords:** fibroblast, cardiac fibrosis, heart failure, regeneration, extracellular matrix

## Abstract

In heart disease patients, myocyte loss or malfunction invariably leads to fibrosis, involving the activation and accumulation of cardiac fibroblasts that deposit large amounts of extracellular matrix. Apart from the vital replacement fibrosis that follows myocardial infarction, ensuring structural integrity of the heart, cardiac fibrosis is largely considered to be maladaptive. Much work has focused on signaling pathways driving the fibrotic response, including TGF-β signaling and biomechanical strain. However, currently there are very limited options for reducing cardiac fibrosis, with most patients suffering from chronic fibrosis. The adult heart has very limited regenerative capacity. However, cardiac regeneration has been reported in humans perinatally, and reproduced experimentally in neonatal mice. Furthermore, model organisms such as the zebrafish are able to fully regenerate their hearts following massive cardiac damage into adulthood. Increasing evidence points to a transient immuno-fibrotic response as being key for cardiac regeneration to occur. The mechanisms at play in this context are changing our views on fibrosis, and could be leveraged to promote beneficial remodeling in heart failure patients. This review summarizes our current knowledge of fibroblast properties associated with the healthy, failing or regenerating heart. Furthermore, we explore how cardiac fibroblast activity could be targeted to assist future therapeutic approaches.

## 1 Introduction

Cardiovascular disease is the leading cause of adult death, and a major cause of disability, worldwide (Benjamin et al., 2019). Its incidence has been on the rise due to our ageing population, sedentary behavior, and poor dietary habits. Cardiovascular diseases are a vast group of disorders of the heart and blood vessels, arising from etiologies as diverse as diabetes, myocarditis, and myocardial infarction, all with a common outcome, the spiral of destruction that is progressive heart failure (HF). A major component driving HF is cardiac fibrosis. The latter is a pathological remodeling process, classically defined as the activation and accumulation of cardiac fibroblasts, resulting in excessive overproduction of fibrous connective tissue (Baudino et al., 2006). Cardiac fibroblasts (CFs) are the cell type chiefly responsible for producing the extracellular matrix (ECM), the dynamic modulatory network comprising a multitude of structural and non-structural macromolecules (Goldsmith et al., 2014). They are emerging as an intriguing cell type to study, regarding both normal cardiac functions, and in response to injury. It is becoming clear that the modulation of fibroblast activity will be key in the future management of HF patients.

Historically, fibroblast were considered to be a highly numerous, if not the most numerous, cell type (Souders et al., 2009). A recent study relying on robust fibroblast markers placed their number at only 11% of total cells in mouse heart, substantially less than previous estimations (Pinto et al., 2016). Zebrafish heart is composed of a far lower proportion of epicardial cells/fibroblasts, at just 1%–2% of all cells (Rolland et al., 2021). Concerning human heart, estimates vary between 10% and 30% of cells, but approaches such as quantification of immune-labelled fibroblasts is less efficient than in mice (Pinto et al., 2016). Diversity in CF proportions among species likely reflect different requirements for ECM content linked to physiological characteristics such as size and mechanical stress, and likely influences response to injury.

Much of the current knowledge on CF function derives from studies focusing on fibrosis and HF. However, recent studies in the field of cardiac repair and regeneration have changed our view on this process. Indeed, both regenerative and non-regenerative responses include inflammatory and proliferative phases, the latter involving fibroblast proliferation and ECM deposition. However, contrary to HF, regeneration is associated with a transient fibrotic response (Sánchez-Iranzo et al., 2018). Exploiting the signaling driving fibrosis resolution could offer new therapeutic possibilities for regulating fibrosis in HF patients. In this review, we discuss properties of fibroblasts and the fibrotic process in HF and regeneration, highlighting common and context-specific features.

## 2 Fibroblasts in the healthy heart

### 2.1 Markers

Fibroblasts have been defined as widely distributed mesenchymal cells that produce extracellular matrix constituents, notably Collagen Type I (Souders et al., 2009). However, the relative abundance, origins and biological properties CFs have remained controversial due to a lack of consensus over definitive markers. Several studies using putative markers such as FSP1 or CD90/thy-1, now known to be expressed by other lineages (Moore-Morris et al., 2016), pointed to endothelial-to-mesenchymal transition and recruitment of circulating progenitors as major sources of fibroblasts in HF (Zeisberg et al., 2007). More recently, the tyrosine kinase receptor, platelet-derived growth factor receptor alpha polypeptide (PDGFRα) and the bHLH transcription factor, Tcf21, have been widely accepted as a comprehensive markers for fibroblasts in both mice and zebrafish (Smith et al., 2011; Acharya et al., 2012; Sánchez-Iranzo et al., 2018) (see Table 1). Furthermore, the development of single cell/nuclei sequencing has revealed that fibroblasts form a clearly identifiable cell-type and validated the specificity of markers such as PDGFRα for the fibroblast lineage in humans (Litviňuková et al., 2020).

**TABLE 1:** Major fibroblast markers, genetic tools and antibodies for their identification and lineage tracing in mice and zebrafish.

Marker	Fibroblast status	Specificity	Expression in other cardiac cell types	Mouse	Zebrafish
Tcf21	All	High	Epicardium	Tcf21^iCre/+^ (Acharya et al., 2011)	tcf21:DsRed2 and tcf21:CreERT2 (Kikuchi et al., 2011)
Pdgfrα	All	High	Epicardium, endocardium	Ab staining (Moore-Morris et al., 2014); PDGFR^αEGFP^ (Hamilton et al., 2003)	*pdgfra:mCitrine; kdrl:mCherry* (Wang et al., 2020a)
ColI*	All	High	Epicardium, activated VSMCs, activated pericytes	coll1a1-GFP (Yata, 2003)	Tg (col1a2:loxP-mCherryNTR) (Sánchez-Iranzo et al., 2018)
Ddr2	All	High	Epicardium	Ab staining (Goldsmith et al., 2004; Banerjee et al., 2007)	N/A
αSMA	Activated/myofibroblast	Low	VSMCs, pericytes	Ab staining (Moore-Morris et al., 2014; Fu et al., 2018); SMA-Cre-ER (T2) (Wendling et al., 2009)	Ab staining (Rolland et al., 2023)
Periostin^a^	Activated/myofibroblast	High	N/A	Postn^*MCM/+*^ (Kanisicak et al., 2016)	*postb-CreERT2* (Sánchez-Iranzo et al., 2018)

^a^ ColI and periostin are secreted ECM, constituents for which reporter lines, but not immunostaining, accurately labels fibroblasts. Ab, antibody. VSMCs, vascular smooth muscle cells.

Fibroblast activation has been explored in common murine models of HF, notably coronary artery ligation and transverse aortic constriction, discussed in more detail below. In response to cardiac injury or stress, fibroblasts undergo activation, including fibroblast-to-myofibroblast transition. As well as for morphological reasoning, the term myofibroblasts was coined due to their *de novo* expression of contractile genes, including ACTA2, which codes for α smooth muscle actin (α-SMA) (Gabbiani et al., 1971). α-SMA expression characterizes myofibroblasts, and allows these cells to physically contribute to scar tissue remodeling, but it is worthwhile noting that it is also used to mark for vascular smooth muscle cells (vSMCs) and is not expressed by all activated fibroblasts (Moore-Morris et al., 2015). Fibroblast activation protein α (FAP), a membrane-bound serine protease, has also been shown to be associated with cardiac myofibroblasts (Tillmanns et al., 2015). Another key marker for myofibroblasts is the matricellular protein periostin, which is associated with a strong fibrotic response after injury (Snider et al., 2009). It is important to note that the use of (secreted) ECM constituents as markers for fibroblasts can lead to mislabeling, which can be overcome at least in animal models by the use of reporter lines (see Table 1).

### 2.2 Origins

During development, the majority of CFs derive from epithelial-to mesenchymal transition of the epicardium, as first shown in the avian system by retroviral tagging of proepicardial progenitors in chick embryos (Mikawa and Gourdie, 1996). PDGFR signaling has been shown to play a key role in this process (Smith et al., 2011). Genetic lineage-tracing methods have confirmed that a majority of fibroblasts derive from epicardium but also revealed that a subset of fibroblasts, enriched in the interventricular septum, are derived from embryonic endocardium (Moore-Morris et al., 2014; Han et al., 2023). Importantly, these developmentally-derived populations give rise to the vast majority of fibroblasts in fibrotic conditions (Ali et al., 2014; Moore-Morris et al., 2014; Han et al., 2023). The endocardium forms the inner most layer of the heart chambers. During mid-gestation, endocardial EMT contributes mesenchymal cells to the forming atrioventricular and semilunar valves (de Lange et al., 2004). A subset of fibroblasts produced during formation of the atrioventircular valves migrate invests the ventricular septum and sub-endocardial myocardium, complementing the distribution of the epicardium-derived fibroblasts (Moore-Morris et al., 2014). Lastly, the cardiac neural crest, a heterogeneous cell population originating from the dorsal neural tube, has been reported to provide a small proportion of fibroblasts to the atria and ventricles (Ali et al., 2014). Hence, in mammalian heart, most fibroblasts are of epicardial origin, but smaller subsets are derived from endocardium and neural crest. Similarly, genetic lineage tracing in zebrafish also showed that fibroblasts are essentially of epicardial origin (Sánchez-Iranzo et al., 2018). Fibrosis, the excessive accumulation of fibroblasts, results from the activation and proliferation of these resident fibroblast lineages (Moore-Morris et al., 2014; Ruiz-Villalba et al., 2015).

## 3 Fibrosis in HF models

HF is defined as a clinical syndrome resulting from structural and/or functional changes resulting in cardiac dysfunction, that can be associated with preserved (HFpEF) or reduced (HFrEF) ejection fraction (EF) (Ponikowski et al., 2016). Multiple disorders and diseases affecting heart development and physiology lead to HF, which is invariably associated with fibrosis of the perivascular and interstitial areas of the myocardium. Murine HF models have been extensively used to explore the cellular and molecular mechanisms driving fibrosis.

### 3.1 Myocardial infarction

Coronary artery ligation is used to induce acute ischemic injury or chronic myocardial infarction (MI) by temporarily or permanently occluding the left anterior descending (LAD) coronary artery (Xu et al., 2014). This results in myocardial ischemia and the gradual replacement of dead myocardial tissue by an infarct scar. The fibroblasts that form the infarct scar have been shown to derive from resident fibroblasts (Ruiz-Villalba et al., 2015; Moore-Morris et al., 2018), as well as *de novo* epicardial-EMT (Zhou et al., 2011). Collagen-GFP^+^;CD45^+^ fibrocytes, circulating cells of hematopoietic origin, have also been observed at the epicardial surface of the heart as a result of the surgical procedure (Moore-Morris et al., 2018). During the infarction process, fibroblasts transition between different states. Notably, scar formation results from fibroblast proliferation and α-SMA^+^ myofibroblast transition during the first week after infarction. Following this, α-SMA expression and collagen production is reduced, as the scar matures (Fu et al., 2018). Importantly, increased left ventricular wall rupture has been linked to reduced cardiac fibroblast activity (Ruiz-Villalba et al., 2020; Murtha et al., 2023).

### 3.2 Transverse aortic constriction

Transverse aortic constriction (TAC) is the most common experimental model used to achieve pressure overload-induced cardiac hypertrophy and HF (Rockman et al., 1991). During the surgical procedure, surgical suture is tied around the transverse aortic arch, resulting in left-sided HF through pressure overload. Initially, depending on the tightness of the aortic ligation, the heart undergoes a hypertrophic compensatory response, that can be associated with a conserved or slightly improved ejection fraction, but ultimately develops signs of HF (Richards et al., 2019). As in the MI model, resident fibroblast populations undergo a proliferative response, expanding in perivascular and interstitial areas (Moore-Morris et al., 2014). Interestingly, resident fibroblasts of endocardial origin have recently been shown to be highly responsive to pressure overload compared with epicardium-derived fibroblasts, and specifically targeting endocardium-derived fibroblasts alleviates pathological remodeling (Han et al., 2023).

## 4 Fibrosis in regeneration models

Regeneration refers to the restoration of normal organ architecture and function following injury ***.*** In many organs, such as the liver or skin, fibrosis is a transient process that naturally recedes during healing. The adult mammalian heart has a very limited regenerative capacity, and most of our understanding of cardiac regenerative biology has come from animal models such as the zebrafish (Hortells et al., 2019). Characterizing the fibrotic process in these models is currently an intense area of research.

### 4.1 Adult teleost fish heart regeneration

The adult zebrafish heart has remarkable regenerative competence, being able to fully regenerate after enduring up to 20% ventricular amputation, a feat in which the adult mammalian heart cannot compare (Poss et al., 2002). Upon cardiac injury, rather than regenerating, mammalian hearts develops chronic fibrosis, a response which is unfortunately maladaptive as it results in permanently altered cardiac form and function, and ultimately, HF (Talman and Ruskoaho, 2016). Conversely, in the zebrafish, fibrosis represents an integrative part of the regenerative response. In zebrafish cardiac regeneration, it is well accepted that new cardiomyocytes derive from pre-existing cardiomyocytes that undergo dedifferentiation, proliferation and redifferentiation (Jopling et al., 2010). Recently, genetic deletion experiments of collagen-producing cells revealed that fibroblast-mediated fibrosis was essential for cardiac regeneration in this model (Sánchez-Iranzo et al., 2018). It is currently unclear to what extent fibroblasts themselves determine the hearts capacity to regenerate, i.e., whether CFs in myocardium with regenerative capacity possess specific properties as compared to CFs in non-regenerative contexts. Interestingly, analysis of the regenerative response in two populations of *Astyanax mexicanus* fish has provided novel insight into this question. Indeed, following apical resection, myocyte proliferation was equivalent in both the non-regenerative cave-dwelling and regenerative surface populations. However, the former presented heightened immune and fibrotic responses, suggesting that modulating non-myocyte behavior will be equally as important as promoting myocyte proliferation to achieve regeneration in HF patients (Stockdale et al., 2018). In zebrafish, single cell RNA-sequencing (scRNA-seq) analysis revealed that ɑSMA was not significantly upregulated in CFs during the regenerative response, in contrast to CFs following myocardial infarction (Rolland et al., 2023). Hence, comparative analyses between models may reveal specific features of fibroblasts that are essential for the regenerative response.

### 4.2 Neonatal mouse heart regeneration

Cardiac regeneration following apical resection has been reported to occur un neonatal mice (1 day postnatal), with resected animals displaying normal structure and function by 3-week of age (Porrello et al., 2011). This recovery has been associated with cardiomyocyte proliferation, as well as a transient fibrotic response (Porrello et al., 2011), similar to what is observed in adult zebrafish (Poss et al., 2002). In a neonatal MI model, a more robust fibrotic response was observed when MI was induced at a non-regenerative stage (P7) compared to the 1 day time point, notably including increased fibroblast proliferation (Wang Z. et al., 2020).

**FIGURE 1:**
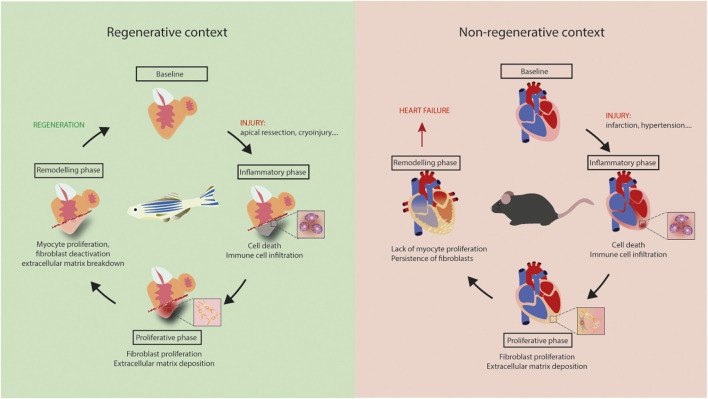
Regeneration *versus* heart failure. Equivalent inflammatory and proliferative phases occur in adult mouse and zebrafish hearts following injury. Lack of myocyte proliferation in mouse heart is associated with the persistence of fibrosis, leading to tissue stiffening, ischemia and, ultimately, heart failure. Regeneration is zebrafish results from myocyte proliferation and gradual resorption of fibrosis.

Fibrotic responses occur in all of these models, but are different in nature. In summary, depending on the context, fibrosis is an essential part of response to insult, as for zebrafish heart regeneration, or a maladaptive process, as in non-ischemic HF patients. Regeneration appears to be dependent on cardiomyocyte proliferation, but also on specific properties of cardiac fibroblasts that support the formation of new myocardium (Figure 1). A better understanding of the signaling regulating fibrosis would likely be beneficial for novel therapeutic approaches aimed at alleviating HF.

## 5 Signaling regulating cardiac fibrosis

A myriad of cell-signaling pathways have been implicated in the activation of fibroblasts. Activated fibroblasts, or myofibroblasts, are known to secrete large amounts of ECM components, including collagens and proteoglycans, that accumulate in interstitial and perivascular areas (Souders et al., 2009). Novel therapeutic targeting of these processes is of intense clinical interest due to the paucity of current treatments available to combat cardiac fibrosis.

Traditional pathways mediating CF activation include transforming growth factor-β (TGF-β), renin-angiotensin-aldosterone system (RAAS), tumour necrosis factor-α (TNFα), and Wnt (Prabhu and Frangogiannis, 2016; Khalil et al., 2017; Schumacher and Naga Prasad, 2018; Działo et al., 2021), whereas more recently studied mediators include endothelin-1, galectin-3, and interleukin 11 (Ho et al., 2012; Wang et al., 2015; Schafer et al., 2017).

Transforming growth factor-beta (TGF-β) proteins bind to cell-surface receptors and regulate key cellular processes, notably by activating Smad proteins that translocate to the nucleus and regulate transcription (Derynck and Zhang, 2003). TGF-β signaling is known to promote ECM deposition and fibrosis (Border and Noble, 1994), and TGF-β neutralization has been shown to prevent CF activation and reduce fibrosis (Kuwahara et al., 2002). More recently, conditional deletion of TGF-β receptors and Smads in CFs markedly reduced fibrosis in a pressure overload model (Khalil et al., 2017). However, TGF-β signaling has also been shown to be essential for cardiac regeneration in zebrafish, as chemical inhibition inhibited recovery from cryoinjury (Chablais and Jazwinska, 2012). Furthermore, TGF-β signaling has been shown to drive zebrafish valve regeneration, notably by promoting mesenchymal progenitor proliferation and differentiation (Bensimon-Brito et al., 2020). Hence, studies focusing on this pathway have revealed that TGF-β signaling is responsible for promoting excessive fibroblast activation in the context of heart disease, but is essential for the fibrotic response required in the context of cardiac regeneration.

Wingless/Int (WNT) signaling plays a key role during development, and involves β-catenin dependent and independent, non-canonical, pathways (Logan and Nusse, 2004). Wnt signaling is notably known to regulate cellular polarity and motility. Conditional deletion of β-catenin in CFs lead to reduced matrix deposition by fibroblasts following pressure overload in mice (Xiang et al., 2017). Canonical WNT signaling has been shown to be required for zebrafish heart regeneration by promoting cardiomyocyte proliferation (Bertozzi et al., 2022). Whether Wnt signaling in fibroblasts is required for scar absorption in this context is currently unclear.

Of late, the little studied interleukin (IL)-11 has captured great interest, being proposed as a newer mediator in the pathogenesis of fibrotic diseases (Fung et al., 2022). IL-11 is a multifunctional member of the IL-6 cytokine family and is known to play a pivotal pro-fibrotic role in various inflammatory diseases (Fung et al., 2022). It was demonstrated in primary human fibroblasts that TGF-β1 is a potent stimulator of IL-11 gene transcription, and inhibiting IL-11 signaling in fibroblasts *in vivo* lead to reduced fibrosis (Schafer et al., 2017). In zebrafish, IL-11/STAT3 signaling has been shown to limit fibrosis and promote cardiac regeneration (Allanki et al., 2021).

Fibroblasts are highly sensitive to mechanical and bio molecular cues, and have been referred to as “sentinel cells” (Smith et al., 1997). Notably, increased stiffness leads to fibroblast-to-myofibroblast transition (van Putten et al., 2016). Knockdown of mechanosensitive channel TRPV4 in CFs has been shown to impair TGF-β1 mediated myofibroblast transition (Adapala et al., 2013). Furthermore, Piezo1 signaling in CFs has been shown to be upstream of pro-inflammatory IL6 secretion (Blythe et al., 2019). Hence, transitions between different fibroblast states are highly dependent on mechanosensing, and further characterizing mechanoreceptor functions in regenerative and non-regenerative contexts may reveal novel therapeutic targets.

## 6 Conclusion/perspectives

Fibrosis has long been considered a pathologic process, and the roles of fibroblasts in disease and regeneration contexts were largely unknown. The identification of specific fibroblast markers, the generation of genetic tools and the development of single cell/nuclei-sequencing have enabled tremendous progress in the characterization of CFs. Moreover, through studies using multiple models of HF and regeneration, it transpires that cardiac fibrosis is a multi-phased process: required in the initial response to insult, but often detrimental in its chronic form. Hence, in the future, regulating rather than inhibiting cardiac fibroblast activity may well be decisive for improving HF patient treatment.

## References

[B1] Acharya A. Baek S. T. Banfi S. Eskiocak B. Tallquist M. D. (2011). Efficient inducible Cre-mediated recombination in Tcf21 cell lineages in the heart and kidney. Genes. (New York, N.Y. 2000) 49 (11), 870–877. 10.1002/dvg.20750 PMC3279154 21432986

[B2] Acharya A. Baek S. T. Huang G. Eskiocak B. Goetsch S. Sung C. Y. (2012). The bHLH transcription factor Tcf21 is required for lineage-specific EMT of cardiac fibroblast progenitors. Dev. Camb. Engl. 139 (12), 2139–2149. 10.1242/dev.079970 PMC3357908 22573622

[B3] Adapala R. K. Thoppil R. J. Luther D. J. Paruchuri S. Meszaros J. G. Chilian W. M. (2013). TRPV4 channels mediate cardiac fibroblast differentiation by integrating mechanical and soluble signals. J. Mol. Cell. Cardiol. 54, 45–52. 10.1016/j.yjmcc.2012.10.016 23142541 PMC3935769

[B4] Ali S. R. Ranjbarvaziri S. Talkhabi M. Zhao P. Subat A. Hojjat A. (2014). Developmental heterogeneity of cardiac fibroblasts does not predict pathological proliferation and activation. Circulation Res. 115 (7), 625–635. 10.1161/CIRCRESAHA.115.303794 25037571

[B5] Allanki S. Strilic B. Scheinberger L. Onderwater Y. L. Marks A. Günther S. (2021). Interleukin-11 signaling promotes cellular reprogramming and limits fibrotic scarring during tissue regeneration. Sci. Adv. 7 (37), eabg6497. 10.1126/sciadv.abg6497 34516874 PMC8442930

[B6] Banerjee I. Fuseler J. W. Price R. L. Borg T. K. Baudino T. A. (2007). Determination of cell types and numbers during cardiac development in the neonatal and adult rat and mouse. Am. J. Physiology-Heart Circulatory Physiology 293 (3), H1883–H1891. 10.1152/ajpheart.00514.2007 17604329

[B7] Baudino T. A. Carver W. Giles W. Borg T. K. (2006). Cardiac fibroblasts: friend or foe? Am. J. Physiology. Heart Circulatory Physiology 291 (3), H1015–H1026. 10.1152/ajpheart.00023.2006 16617141

[B8] Benjamin E. J. Muntner P. Alonso A. Bittencourt M. S. Callaway C. W. Carson A. P. (2019). Heart disease and stroke statistics-2019 update: a report from the American heart association. Circulation 139 (10), e56–e528. 10.1161/CIR.0000000000000659 30700139

[B9] Bensimon-Brito A. Ramkumar S. Boezio G. L. M. Guenther S. Kuenne C. Helker C. S. M. (2020). TGF-Β signaling promotes tissue formation during cardiac valve regeneration in adult zebrafish. Dev. Cell 52 (1), 9–20. 10.1016/j.devcel.2019.10.027 31786069

[B10] Bertozzi A. Wu C.-C. Hans S. Brand M. Weidinger G. (2022). Wnt/β-catenin signaling acts cell-autonomously to promote cardiomyocyte regeneration in the zebrafish heart. Dev. Biol. 481, 226–237. 10.1016/j.ydbio.2021.11.001 34748730

[B11] Blythe N. M. Muraki K. Ludlow M. J. Stylianidis V. Gilbert H. T. J. Evans E. L. (2019). Mechanically activated Piezo1 channels of cardiac fibroblasts stimulate p38 mitogen-activated protein kinase activity and interleukin-6 secretion. J. Biol. Chem. 294 (46), 17395–17408. 10.1074/jbc.RA119.009167 31586031 PMC6873183

[B12] Border W. A. Noble N. A. (1994). Transforming growth factor beta in tissue fibrosis. N. Engl. J. Med. 331 (19), 1286–1292. 10.1056/NEJM199411103311907 7935686

[B13] Chablais F. Jazwinska A. (2012). The regenerative capacity of the zebrafish heart is dependent on TGFβ signaling. Dev. Camb. Engl. 139 (11), 1921–1930. 10.1242/dev.078543 22513374

[B14] de Lange F. J. Moorman A. F. M. Anderson R. H. Männer J. Soufan A. T. de Gier-de Vries C. (2004). Lineage and morphogenetic analysis of the cardiac valves. Circulation Res. 95 (6), 645–654. 10.1161/01.RES.0000141429.13560.cb 15297379

[B15] Derynck R. Zhang Y. E. (2003). Smad-dependent and Smad-independent pathways in TGF-beta family signalling. Nature 425 (6958), 577–584. 10.1038/nature02006 14534577

[B16] Działo E. Czepiel M. Tkacz K. Siedlar M. Kania G. Błyszczuk P. (2021). WNT/β-Catenin signaling promotes TGF-β-mediated activation of human cardiac fibroblasts by enhancing IL-11 production. Int. J. Mol. Sci. 22 (18), 10072. 10.3390/ijms221810072 34576234 PMC8468519

[B17] Fu X. Khalil H. Kanisicak O. Boyer J. G. Vagnozzi R. J. Maliken B. D. (2018). Specialized fibroblast differentiated states underlie scar formation in the infarcted mouse heart. J. Clin. Investigation 128 (5), 2127–2143. 10.1172/JCI98215 PMC5957472 29664017

[B18] Fung K. Y. Louis C. Metcalfe R. D. Kosasih C. C. Wicks I. P. Griffin M. D. W. (2022). Emerging roles for IL-11 in inflammatory diseases. Cytokine 149, 155750. 10.1016/j.cyto.2021.155750 34689057

[B19] Gabbiani G. Ryan G. B. Majne G. (1971). Presence of modified fibroblasts in granulation tissue and their possible role in wound contraction. Experientia 27 (5), 549–550. 10.1007/BF02147594 5132594

[B20] Goldsmith E. C. Bradshaw A. D. Zile M. R. Spinale F. G. (2014). Myocardial fibroblast-matrix interactions and potential therapeutic targets. J. Mol. Cell. Cardiol. 70, 92–99. 10.1016/j.yjmcc.2014.01.008 24472826 PMC4005609

[B21] Goldsmith E. C. Hoffman A. Morales M. O. Potts J. D. Price R. L. McFadden A. (2004). Organization of fibroblasts in the heart. Dev. Dyn. 230 (4), 787–794. 10.1002/dvdy.20095 15254913

[B22] Hamilton T. G. Klinghoffer R. A. Corrin P. D. Soriano P. (2003). Evolutionary divergence of platelet-derived growth factor alpha receptor signaling mechanisms. Mol. Cell. Biol. 23 (11), 4013–4025. 10.1128/MCB.23.11.4013-4025.2003 12748302 PMC155222

[B23] Han M. Liu Z. Liu L. Huang X. Wang H. Pu W. (2023). Dual genetic tracing reveals a unique fibroblast subpopulation modulating cardiac fibrosis. Nat. Genet. 55, 665–678. 10.1038/s41588-023-01337-7 36959363

[B24] Ho J. E. Liu C. Lyass A. Courchesne P. Pencina M. J. Vasan R. S. (2012). Galectin-3, a marker of cardiac fibrosis, predicts incident heart failure in the community. J. Am. Coll. Cardiol. 60 (14), 1249–1256. 10.1016/j.jacc.2012.04.053 22939561 PMC3512095

[B25] Hortells L. Johansen A. K. Z. Yutzey K. E. (2019). Cardiac fibroblasts and the extracellular matrix in regenerative and nonregenerative hearts. J. Cardiovasc. Dev. Dis. 6 (3), 29. 10.3390/jcdd6030029 31434209 PMC6787677

[B26] Jopling C. Sleep E. Raya M. Martí M. Raya A. Izpisúa Belmonte J. C. (2010). Zebrafish heart regeneration occurs by cardiomyocyte dedifferentiation and proliferation. Nature 464 (7288), 606–609. 10.1038/nature08899 20336145 PMC2846535

[B27] Kanisicak O. Khalil H. Ivey M. J. Karch J. Maliken B. D. Correll R. N. (2016). Genetic lineage tracing defines myofibroblast origin and function in the injured heart. Nat. Commun. 7, 12260. 10.1038/ncomms12260 27447449 PMC5512625

[B28] Khalil H. Kanisicak O. Prasad V. Correll R. N. Fu X. Schips T. (2017). Fibroblast-specific TGF-β-Smad2/3 signaling underlies cardiac fibrosis. J. Clin. Investigation 127 (10), 3770–3783. 10.1172/JCI94753 PMC5617658 28891814

[B29] Kikuchi K. Gupta V. Wang J. Holdway J. E. Wills A. A. Fang Y. (2011). *tcf21+* epicardial cells adopt non-myocardial fates during zebrafish heart development and regeneration. Development 138 (14), 2895–2902. 10.1242/dev.067041 21653610 PMC3119303

[B30] Kuwahara F. Kai H. Tokuda K. Kai M. Takeshita A. Egashira K. (2002). Transforming growth factor-beta function blocking prevents myocardial fibrosis and diastolic dysfunction in pressure-overloaded rats. Circulation 106 (1), 130–135. 10.1161/01.cir.0000020689.12472.e0 12093782

[B31] Litviňuková M. Talavera-López C. Maatz H. Reichart D. Worth C. L. Lindberg E. L. (2020). Cells of the adult human heart. Nature 588 (7838), 466–472. 10.1038/s41586-020-2797-4 32971526 PMC7681775

[B32] Logan C. Y. Nusse R. (2004). The Wnt signaling pathway in development and disease. Annu. Rev. Cell Dev. Biol. 20, 781–810. 10.1146/annurev.cellbio.20.010403.113126 15473860

[B33] Mikawa T. Gourdie R. G. (1996). Pericardial mesoderm generates a population of coronary smooth muscle cells migrating into the heart along with ingrowth of the epicardial organ. Dev. Biol. 174 (2), 221–232. 10.1006/dbio.1996.0068 8631495

[B34] Moore-Morris T. Cattaneo P. Guimarães-Camboa N. Bogomolovas J. Cedenilla M. Banerjee I. (2018). Infarct fibroblasts do not derive from bone marrow lineages. Circulation Res. 122 (4), 583–590. 10.1161/CIRCRESAHA.117.311490 29269349 PMC5815911

[B35] Moore-Morris T. Cattaneo P. Puceat M. Evans S. M. (2016). Origins of cardiac fibroblasts. J. Mol. Cell. Cardiol. 91, 1–5. 10.1016/j.yjmcc.2015.12.031 26748307 PMC4764439

[B36] Moore-Morris T. Guimarães-Camboa N. Banerjee I. Zambon A. C. Kisseleva T. Velayoudon A. (2014). Resident fibroblast lineages mediate pressure overload-induced cardiac fibrosis. J. Clin. Investigation 124 (7), 2921–2934. 10.1172/JCI74783 PMC4071409 24937432

[B37] Moore-Morris T. Guimarães-Camboa N. Yutzey K. E. Pucéat M. Evans S. M. (2015). Cardiac fibroblasts: from development to heart failure. J. Mol. Med. Berlin, Ger. 93 (8), 823–830. 10.1007/s00109-015-1314-y PMC4512919 26169532

[B38] Murtha L. A. Hardy S. A. Mabotuwana N. S. Bigland M. J. Bailey T. Raguram K. (2023). Fibulin-3 is necessary to prevent cardiac rupture following myocardial infarction. Sci. Rep. 13 (1), 14995. 10.1038/s41598-023-41894-9 37696945 PMC10495317

[B39] Pinto A. R. Ilinykh A. Ivey M. J. Kuwabara J. T. D’Antoni M. L. Debuque R. (2016). Revisiting cardiac cellular composition. Circulation Res. 118 (3), 400–409. 10.1161/CIRCRESAHA.115.307778 26635390 PMC4744092

[B40] Ponikowski P. Voors A. A. Anker S. D. Bueno H. Cleland J. G. F. Coats A. J. S. (2016). 2016 ESC Guidelines for the diagnosis and treatment of acute and chronic heart failure: the Task Force for the diagnosis and treatment of acute and chronic heart failure of the European Society of Cardiology (ESC)Developed with the special contribution of the Heart Failure Association (HFA) of the ESC. Eur. Heart J. 37 (27), 2129–2200. 10.1093/eurheartj/ehw128 27206819

[B41] Porrello E. R. Mahmoud A. I. Simpson E. Hill J. A. Richardson J. A. Olson E. N. (2011). Transient regenerative potential of the neonatal mouse heart. Sci. (New York, N.Y.) 331 (6020), 1078–1080. 10.1126/science.1200708 PMC3099478 21350179

[B42] Poss K. D. Wilson L. G. Keating M. T. (2002). Heart regeneration in zebrafish. Sci. (New York, N.Y.) 298 (5601), 2188–2190. 10.1126/science.1077857 12481136

[B43] Prabhu S. D. Frangogiannis N. G. (2016). The biological basis for cardiac repair after myocardial infarction: from inflammation to fibrosis. Circulation Res. 119 (1), 91–112. 10.1161/CIRCRESAHA.116.303577 27340270 PMC4922528

[B44] Richards D. A. Aronovitz M. J. Calamaras T. D. Tam K. Martin G. L. Liu P. (2019). Distinct phenotypes induced by three degrees of transverse aortic constriction in mice. Sci. Rep. 9 (1), 5844. 10.1038/s41598-019-42209-7 30971724 PMC6458135

[B45] Rockman H. A. Ross R. S. Harris A. N. Knowlton K. U. Steinhelper M. E. Field L. J. (1991). Segregation of atrial-specific and inducible expression of an atrial natriuretic factor transgene in an *in vivo* murine model of cardiac hypertrophy. Proc. Natl. Acad. Sci. U. S. A. 88 (18), 8277–8281. 10.1073/pnas.88.18.8277 1832775 PMC52490

[B46] Rolland L. Harrington A. Faucherre A. Abaroa J. M. Gangatharan G. Gamba L. (2023). The regenerative response of cardiac interstitial cells. J. Mol. Cell Biol. 14 (10), mjac059. 10.1093/jmcb/mjac059 36271843 PMC10068904

[B47] Rolland L. Harrington A. Faucherre A. Gangatharan G. Gamba L. Severac D. (2021). The regenerative response of cardiac interstitial cells. bioRxiv 2021, 465720. 10.1101/2021.10.25.465720 PMC10068904 36271843

[B48] Ruiz-Villalba A. Romero J. P. Hernández S. C. Vilas-Zornoza A. Fortelny N. Castro-Labrador L. (2020). Single-cell RNA sequencing analysis reveals a crucial role for CTHRC1 (collagen triple helix repeat containing 1) cardiac fibroblasts after myocardial infarction. Circulation 142 (19), 1831–1847. 10.1161/CIRCULATIONAHA.119.044557 32972203 PMC7730974

[B49] Ruiz-Villalba A. Simón A. M. Pogontke C. Castillo M. I. Abizanda G. Pelacho B. (2015). Interacting resident epicardium-derived fibroblasts and recruited bone marrow cells form myocardial infarction scar. J. Am. Coll. Cardiol. 65 (19), 2057–2066. 10.1016/j.jacc.2015.03.520 25975467

[B50] Sánchez-Iranzo H. Galardi-Castilla M. Sanz-Morejón A. González-Rosa J. M. Costa R. Ernst A. (2018). Transient fibrosis resolves via fibroblast inactivation in the regenerating zebrafish heart. Proc. Natl. Acad. Sci. U. S. A. 115 (16), 4188–4193. 10.1073/pnas.1716713115 29610343 PMC5910827

[B51] Schafer S. Viswanathan S. Widjaja A. A. Lim W.-W. Moreno-Moral A. DeLaughter D. M. (2017). IL-11 is a crucial determinant of cardiovascular fibrosis. Nature 552 (7683), 110–115. 10.1038/nature24676 29160304 PMC5807082

[B52] Schumacher S. M. Naga Prasad S. V. (2018). Tumor necrosis factor-α in heart failure: an updated review. Curr. Cardiol. Rep. 20 (11), 117. 10.1007/s11886-018-1067-7 30259192 PMC6311126

[B53] Smith C. L. Baek S. T. Sung C. Y. Tallquist M. D. (2011). Epicardial-derived cell epithelial-to-mesenchymal transition and fate specification require PDGF receptor signaling. Circulation Res. 108 (12), e15–e26. 10.1161/CIRCRESAHA.110.235531 21512159 PMC3134964

[B54] Smith R. S. Smith T. J. Blieden T. M. Phipps R. P. (1997). Fibroblasts as sentinel cells. Synthesis of chemokines and regulation of inflammation. Am. J. Pathology 151 (2), 317–322. PMC1858004 9250144

[B55] Snider P. Standley K. N. Wang J. Azhar M. Doetschman T. Conway S. J. (2009). Origin of cardiac fibroblasts and the role of periostin. Circulation Res. 105 (10), 934–947. 10.1161/CIRCRESAHA.109.201400 19893021 PMC2786053

[B56] Souders C. A. Bowers S. L. K. Baudino T. A. (2009). Cardiac fibroblast: the renaissance cell. Circulation Res. 105 (12), 1164–1176. 10.1161/CIRCRESAHA.109.209809 19959782 PMC3345531

[B57] Stockdale W. T. Lemieux M. E. Killen A. C. Zhao J. Hu Z. Riepsaame J. (2018). Heart regeneration in the Mexican cavefish. Cell Rep. 25 (8), 1997–2007. 10.1016/j.celrep.2018.10.072 30462998 PMC6280125

[B58] Talman V. Ruskoaho H. (2016). Cardiac fibrosis in myocardial infarction-from repair and remodeling to regeneration. Cell Tissue Res. 365 (3), 563–581. 10.1007/s00441-016-2431-9 27324127 PMC5010608

[B59] Tillmanns J. Hoffmann D. Habbaba Y. Schmitto J. D. Sedding D. Fraccarollo D. (2015). Fibroblast activation protein alpha expression identifies activated fibroblasts after myocardial infarction. J. Mol. Cell. Cardiol. 87, 194–203. 10.1016/j.yjmcc.2015.08.016 26319660

[B60] van Putten S. Shafieyan Y. Hinz B. (2016). Mechanical control of cardiac myofibroblasts. J. Mol. Cell. Cardiol. 93, 133–142. 10.1016/j.yjmcc.2015.11.025 26620422

[B61] Wang G. Muhl L. Padberg Y. Dupont L. Peterson-Maduro J. Stehling M. (2020a). Specific fibroblast subpopulations and neuronal structures provide local sources of Vegfc-processing components during zebrafish lymphangiogenesis. Nat. Commun. 11 (1), 2724. 10.1038/s41467-020-16552-7 32483144 PMC7264274

[B62] Wang X. Guo Z. Ding Z. Khaidakov M. Lin J. Xu Z. (2015). Endothelin-1 upregulation mediates aging-related cardiac fibrosis. J. Mol. Cell. Cardiol. 80, 101–109. 10.1016/j.yjmcc.2015.01.001 25584774

[B63] Wang Z. Cui M. Shah A. M. Tan W. Liu N. Bassel-Duby R. (2020b). Cell-type-specific gene regulatory networks underlying murine neonatal heart regeneration at single-cell resolution. Cell Rep. 33 (10), 108472. 10.1016/j.celrep.2020.108472 33296652 PMC7774872

[B64] Wendling O. Bornert J.-M. Chambon P. Metzger D. (2009). Efficient temporally-controlled targeted mutagenesis in smooth muscle cells of the adult mouse. Genes. (New York, N.Y. 2000) 47 (1), 14–18. 10.1002/dvg.20448 18942088

[B65] Xiang F.-L. Fang M. Yutzey K. E. (2017). Loss of β-catenin in resident cardiac fibroblasts attenuates fibrosis induced by pressure overload in mice. Nat. Commun. 8 (1), 712. 10.1038/s41467-017-00840-w 28959037 PMC5620049

[B66] Xu Z. Alloush J. Beck E. Weisleder N. (2014). A murine model of myocardial ischemia-reperfusion injury through ligation of the left anterior descending artery. J. Vis. Exp. JoVE 86, 51329. 10.3791/51329 PMC4080806 24747599

[B67] Yata Y. Scanga A. Gillan A. Yang L. Reif S. Breindl M. (2003). DNase I-hypersensitive sites enhance alpha1(I) collagen gene expression in hepatic stellate cells. Hepatology 37 (2), 267–276. 10.1053/jhep.2003.50067 12540776

[B68] Zeisberg E. M. Tarnavski O. Zeisberg M. Dorfman A. L. McMullen J. R. Gustafsson E. (2007). Endothelial-to-mesenchymal transition contributes to cardiac fibrosis. Nat. Med. 13 (8), 952–961. 10.1038/nm1613 17660828

[B69] Zhou B. Honor L. B. He H. Ma Q. Oh J.-H. Butterfield C. (2011). Adult mouse epicardium modulates myocardial injury by secreting paracrine factors. J. Clin. Investigation 121 (5), 1894–1904. 10.1172/JCI45529 PMC3083761 21505261

